# A Track Geometry Measuring System Based on Multibody Kinematics, Inertial Sensors and Computer Vision

**DOI:** 10.3390/s21030683

**Published:** 2021-01-20

**Authors:** José L. Escalona, Pedro Urda, Sergio Muñoz

**Affiliations:** 1 Department of Mechanical and Manufacturing Engineering, University of Seville, 41092 Seville, Spain; purda@us.es; 2 Department of Materials and Transportation Engineering, University of Seville, 41092 Seville, Spain; sergiomunoz@us.es

**Keywords:** rail vehicles, track irregularities, multibody dynamics, inertial sensors, computer vision

## Abstract

This paper describes the kinematics used for the calculation of track geometric irregularities of a new *Track Geometry Measuring System* (TGMS) to be installed in railway vehicles. The TGMS includes a computer for data acquisition and process, a set of sensors including an *inertial measuring unit* (IMU, 3D gyroscope and 3D accelerometer), two video cameras and an encoder. The kinematic description, that is borrowed from the multibody dynamics analysis of railway vehicles used in computer simulation codes, is used to calculate the relative motion between the vehicle and the track, and also for the computer vision system and its calibration. The multibody framework is thus used to find the formulas that are needed to calculate the track irregularities (gauge, cross-level, alignment and vertical profile) as a function of sensor data. The TGMS has been experimentally tested in a 1:10 scaled vehicle and track specifically designed for this investigation. The geometric irregularities of a 90 m-scale track have been measured with an alternative and accurate method and the results are compared with the results of the TGMS. Results show a good agreement between both methods of calculation of the geometric irregularities.

## 1. Introduction

Measurement of track geometry irregularities is a fundamental task in railway track maintenance with immense economic significance worldwide. Geometric irregularity is the most important track feature affecting safety and comfort of the rail transport. There are two different equipment that rail administrations and infrastructure managers use for track irregularity measurement: man-driven *rail track trolleys* (RTT) and *track recording vehicles* (TRV), also called *laboratory vehicles* or *inspection vehicles*. There are many companies that manufacture both types of equipment. The technology behind the RTT is simple and accurate. The relative irregularities, this is, gauge and cross-level are measured with a distance sensor, like an LVDT, and an inclinometer, respectively. The absolute irregularities, alignment and longitudinal profile require and absolute positioning system, like a total station or a very accurate GNSS. The technology used in TRV is more varied. Essentially, there are two technologies [1]: versine measurement systems (VMS), also called chord-based measurement systems and inertial measurement systems (IMS) or optical-inertial measurement systems. In addition to the sensors used, the main difference between these two methods is on the reference kinematics. The VMS use a physical reference, the chord, that can be a solid bar or a laser beam, while the IMS use an inertial reference frame or, in other words, the frame does not physically exist. The existence of the physical reference is an important advantage of the VMS that improves its accuracy. The main drawback of the VMS is that only irregularities with wave-lengths shorter than the length of the physical reference can be measured. A purely inertial IMS, as those based on accelerometer measurements, is based on the premise that the measuring device follows a trajectory that is parallel to one of the rails or the track centerline. This is in practice very difficult to obtain, or even impossible, if one wants to get the four irregularities with a single device. However, the most serious problem of the IMS is not that but the need to integrate the sensor signals in time to get the irregularities. The resulting accuracy depends very much on the filtering and other signal processing techniques that are needed to avoid the signal drift due to the integration.

The scientific foundation of the VMS is based on simple kinematics that only requires the measurements of distance sensors. The main challenge is to find the transfer function between the chord measurements and the vertical and lateral track irregularities [1,2]. The kinematics of the IMS is much more involved, due to the use of the inertial frame, as mentioned previously, and the use of different sensors (distance sensors, optical and inertial) requiring sensor fusion algorithms. To the authors’ best knowledge, the description of the TRV used in the early eighties in British Rail by Lewis [3] remains the most complete description of the kinematics and signal processing behind the track geometry measurement with IMS. This paper tries to fill this gap. In a paper by Escalona [4], the calculation of the track vertical geometry using inertial sensors mounted on an un-suspended bogie was fully described, including analytical relationships, transfer functions, sensor fusion algorithms and signal processing. However, the method was valid only for the vertical geometry and the results were not experimentally validated.

Despite the existence of different measuring equipment, there is a need for improvement of the technology in track geometry inspection. RRT are much cheaper than TRV (small rail administrations cannot afford TRV) but much slower also. As a result, the frequency of track geometry inspection is not as high as desired. Due to this reason, there is an increasing tendency in the railway industry and research teams to developed track geometry measuring systems (TGMS) to be installed in in-service vehicles. That way, the frequency of the inspection is increased, the vehicle response to the irregularity can be measured simultaneously, and the time-evolution of the irregularities can be followed closely. However, what the sensors can get in an in-service vehicle, in general, is not a measurement of the irregularity but an estimation. There are two approaches: purely kinematic or model-based methods. Weston et al. published a set of papers [5,6,7] showing the measurement of vertical and lateral track irregularities using accelerometers and gyroscopes mounted in the bogie frame and a very simple kinematic model of the vehicle motion. Although these papers do not show results in frequency domain, the measured irregularities could only be relatively accurate for low frequencies, because the bogie frame does not follow the high frequency irregularities. Lee et al. [8] presented a Kalman filter data fusion approach based on accelerometer signals mounted on the body frame and the axle-box. Tsai et al. [9,10] presented a fast inspection technique based on the Hilbert–Huang Transform, that is a very useful time–frequency analysis technique, applied to the signal of an accelerometer mounted on the axle-box of an in-service car. Both methods that are based on axle-box-mounted accelerometers are only valid to measure vertical track irregularities. Tsumashima et al. [11] estimated the vertical track geometry using the accelerations measured in the car-body of the Japanese Shinkansen using a Kalman filter based on a very basic car model. In a more recent work, Tsumashima [12] has develop a method to detect and isolate track faults that are later classified using machine learning techniques. Tsumashima and Hirose [13] have applied the time–frequency Hilbert–Huang Transform method to identify track faults using the measured car-body acceleration as the input.

This paper describes a TGMS based on inertial sensors and computer vision that can be installed in in-service vehicles. The 3D kinematics used for the geometry measurements considers a general track design geometry, and the assumed kinematic approximations and simplifications are fully described. As a result of the background of the authors in on the development of multibody dynamics models for railway simulations [14,15,16], the presented kinematic equations follow the multibody formalism. The kinematic equations used for the computer vision are adapted to this formalism. The sensor fusion algorithms and signal processing tools used to find the track irregularities are explained also. The presented TGMS has been built, tested and validated in an experimental scale track facility. Compared with the existing IMS methods that are implemented in the industry, the contribution of the method presented in this paper lies in the description of the methodology and the experimental validation. To the authors’ best knowledge, the algorithms used to turn the sensors data into measured irregularities have not been described in the scientific literature, or they are described using an over-simplified track geometry, for example, assuming that the track is straight. This information is not provided by the companies that work in the important business of track inspection. In addition, an experimental proof of the accuracy of the equipment used for this task in not generally provided by these companies. This paper provides a full description of the signal processing that can be used to turn the sensors data into track irregularities. As shown below, the kinematic analysis needed to this end is not straightforward. This paper also provides an experimental validation of the measured irregularities. Authors do not claim that the presented method is more accurate or more efficient than existing methods, simply because the data are not accessible to compare.

This paper is organized as follows: Section 2 describes the sensors needed and the main features of the TGMS. Section 3 explains the kinematics of the irregular track and the rail vehicles based on the multibody dynamics formalism. Section 4 applies this formalism to the kinematics of the computer vision with the pin-hole camera model. Section 5 explains the method that is used to find the relative position of the rail cross-sections with respect to the moving vehicle using the computer vision data. Section 6 shows the kinematic relationships that are used to find the four track irregularities: gauge variation, cross-level, alignment and vertical profile, as a function of the sensors data. Section 7 describes a Kalman filter that is used to calculate the TGMS relative orientation with respect to the track. Section 8 describes an odometry algorithm that is used to locate at any instant the vehicle along the track. Section 9 explains the method that is used to find the relative trajectory of the TGMS with respect to the track centerline. The content of Section 5, Section 6, Section 7, Section 8 and Section 9 includes all the calculations needed to obtain the track irregularities out of the sensors data. Finally, Section 10 shows the experimental setup that has been used to build and validate the TGMS system and Section 11 compares the measured irregularities with accurate reference values. Summary and conclusions are given in Section 12.

## 2. Description of the TGMS

**Figure 1:**
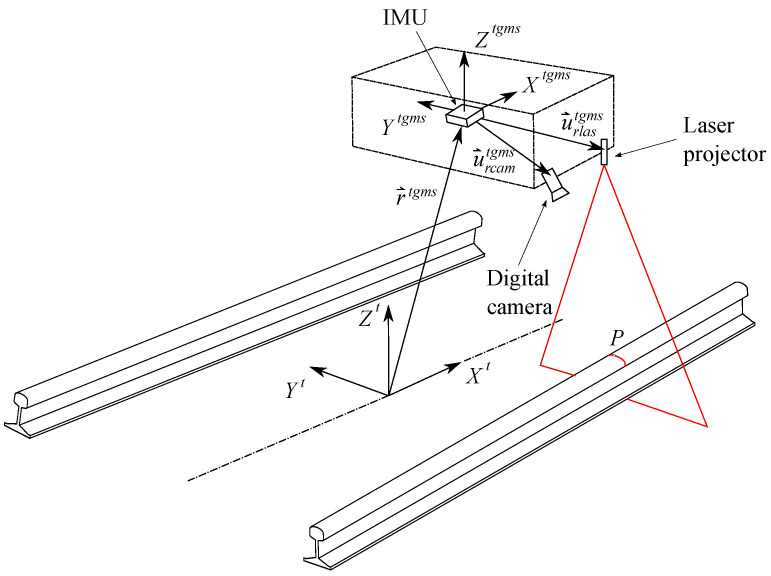
Kinematics of the Track Geometry Measuring System (TGMS) installed in a vehicle moving along the track.

The TGMS sketched in Figure 1 (only right-side equipment in shown here) comprises:

1. Two video cameras.
2. Two laser line-projectors.
3. An IMU.
4. A rotary encoder.

It is important that the cameras, lasers and IMU are installed in a solid that can be considered as a rigid body when moving with the vehicle. The lasers and cameras must be equipped with orientation mechanisms that can be fully locked when the TGMS is working. The laser projectors draw red lines (when using a red laser) in the rail-heads (one on the left, one on the right) that are filmed by the video cameras. The information provided by the position and orientation of the red lines in the rails, together with the acceleration and angular velocity acquired with the IMU, are used to find the track geometry irregularities.

The main features of the proposed system are:

1. It is capable to measure track alignment, vertical profile, cross-level, gauge, twist and rail-head profile using non-contact technology.
2. It can be installed in in-service vehicles. It is compact and low cost. Provided that the equipment sees the rail heads when the vehicle is moving, it can be installed in any body of the vehicle: at the wheelsets level, above primary suspension (bogie frame) or above the secondary suspension (car body).

Measuring devices must be selected taking into account the dusty environment under the train, affecting mainly the computer vision system, and the high level of accelerations that they will be subjected to. Protecting the video cameras and laser projectors may be solved, in part, locating them in a closed box with a polycarbonate window that must be cleaned frequently. Regarding the high level of accelerations, their expected value depends on the solid where the equipment is installed. The most critical body would be the axle-box (wheelset level) where the vertical accelerations can reach 100 g in high velocity trains. Reducing the possibility of damaging the measuring devices due to excessive accelerations is achieved if the equipment is installed at the car-body level.

## 3. Kinematics of the Irregular Track and the Railway Vehicle

This section includes the kinematic description of the rail geometry as a combination of a design geometry and the irregularities, and the kinematic description of an arbitrary body moving along the track, like the TGMS. Before presenting the kinematics, the different frames that are used and the nomenclature used to describe vectors, matrices and their components are described.

### 3.1. Frames of Reference

**Figure 2:**
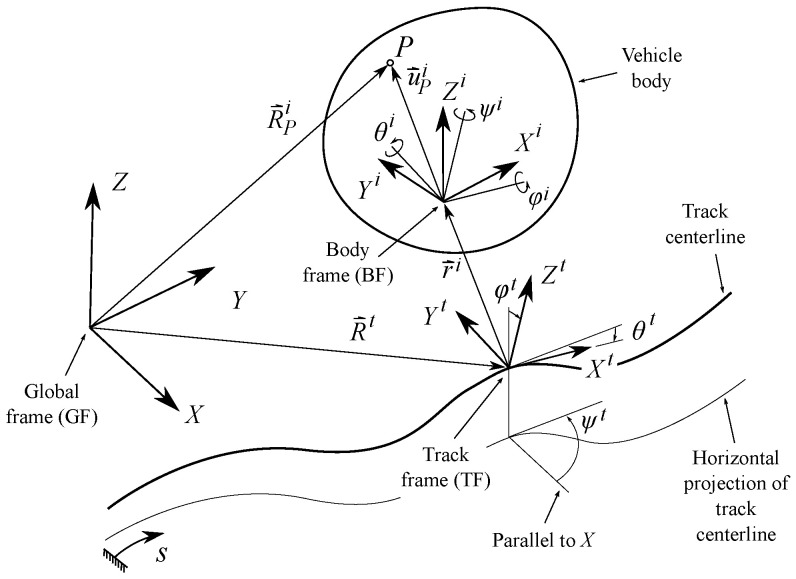
Kinematics of a body moving along the track.

**Figure 3:**
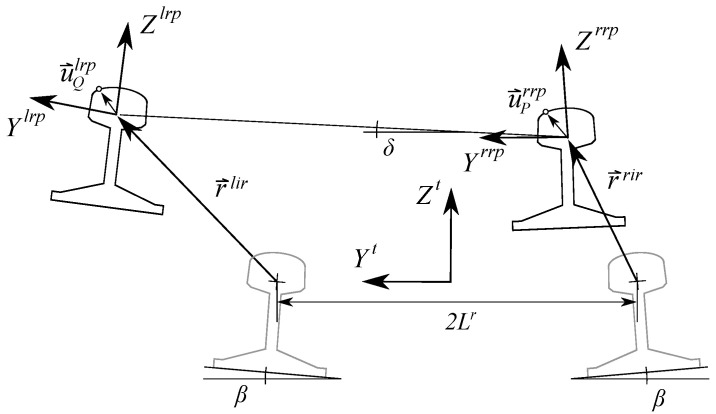
Kinematics of the irregular track cross-section.

As shown in Figure 2 and Figure 3, four different frames are used in railroad kinematics:

1. The inertial and global frame (GF) <X,Y,Z>. It is a frame fixed in space.
2. The track frame (TF) $<X^{t},Y^{t},Z^{t}>$. It is not a single frame but a field defined for each value of the arc-length coordinate along the track *s*. The position $R^{t}\left(s\right)$ and orientation matrix $A^{t}\left(s\right)$ of the TF with respect to the GF are functions of an arc-length coordinate *s* along the center line of the design track (without irregularities). These functions are implemented computationally in a track preprocessor.
3. The body frame (BF) $<X^{i},Y^{i},Z^{i}>$ of each body *i*. It is a frame rigidly attached to the body. In this document, the body *i* is the TGMS. The body frame of the TGMS is denoted as $<X^{tgms},Y^{tgms},Z^{tgms}>$
4. The rail profile frames. Left rail-profile frame (LRP), $<X^{lrp},Y^{lrp},Z^{lrp}>$, and right-profile frame (RRP), $<X^{rrp},Y^{rrp},Z^{rrp}>$. These frames are not unique frames but fields defined for each value of the arc-length coordinate along the track *s*. These frames are rigidly attached to the rail-heads.

The definition of the TF is such that the $X^{t}$ axis is tangent to the track design centerline, the $Y^{t}$ axis is perpendicular to $X^{t}$ and connects the origin $O^{lrp}$ of the LRP and the origin $O^{rrp}$ of the RRP in the design track geometry (with no track irregularities) and the $Z^{t}$ axis is perpendicular to both $X^{t}$ and $Y^{t}$. Therefore, the TF is not the Frenet frame of the design track centerline. Each body *i* moving along the track has an associated TF at each instant of time. Its position and orientation can be obtained substituting the position of the body along the track, $s^{i}\left(t\right)$, in the functions $R^{t}\left(s\right)$ and $A^{t}\left(s\right)$ that are explained in next section. More details about the rail kinematics and the nomenclature used in this work for vectors and rotation matrices can be found in [17].

### 3.2. Kinematics of the Design Track Centerline

Track geometry is the superposition of the design geometry and the irregularities. The components of the absolute position vector of an arbitrary point on the design track centerline with respect to an inertial and global frame is a function of the arc-length *s*, as follows:

(1) $$R^{t}\left(s\right)=\left[\begin{array}{c} R_{x}^{t}\left(s\right)\\ R_{y}^{t}\left(s\right)\\ R_{z}^{t}\left(s\right) \end{array}\right]$$

where $R^{t}$ contains the components of vector ${\vec{R}}^{t}$ shown in Figure 2. The geometry of the track centerline 3D-curve is defined by the horizontal profile and the vertical profile. Both profiles are defined in the rail industry using sections of variable length. Points between two sections are called vertices. Horizontal profile vertices do not necessary coincide with vertical profile vertices. Horizontal profile includes three types of sections: tangent (straight), curve (circular) and transitions (clothoid). Vertical profile includes two types of sections: constant-slope (straight) and transitions (cubic).

At each track section, the track centerline geometry is characterized by the following geometric values:

- Horizontal curvature: $\rho_{h}$.
- Vertical curvature: $\rho_{v}$.
- Twist curvature: $\rho_{tw}$.
- Spatial-derivative of horizontal curvature: ${\rho_{h}}^{\prime }$.
- Vertical slope: $\alpha_{v}$.

Figure 2 shows the TF $<X^{t},Y^{t},Z^{t}>$ associated with the track centerline at each value of *s*. The orientation of the TF with respect to a GF can be measured with the Euler angles $\psi^{t}$ (azimuth or heading angle), $\theta^{\;t}$ (vertical slope, positive when downwards in the forward direction) and $\varphi^{\;t}$ (cant or superelevationangle). The rotation matrix from the TF to the GF is given by:

(2) $$A^{t}\left(s\right)=\left[\begin{array}{ccc} c\theta^{\;t}c\psi^{t} & s\varphi^{t}s\theta^{\;t}c\psi^{t}-c\varphi^{t}s\psi^{t} & s\varphi^{t}s\psi^{t}+c\varphi^{t}s\theta^{\;t}c\psi^{t}\\ c\theta^{\;t}s\psi^{t} & c\varphi^{t}c\psi^{t}+s\varphi^{t}s\theta^{\;t}s\psi^{t} & c\varphi^{t}s\theta^{\;t}s\psi^{t}-s\varphi^{t}c\psi^{t}\\ -s\theta^{\;t} & s\varphi^{t}c\theta^{\;t} & c\varphi^{t}c\theta^{\;t} \end{array}\right]$$

The azimuth $\psi^{t}$ can have an arbitrary value, however, the slope $\theta^{\;t}$ and cant $\varphi^{\;t}$ angles can be considered as small angles, such that the rotation matrix from the TF to the GF can be approximated to:

(3) $$A^{t}\left(s\right)≃\left[\begin{array}{ccc} c\psi^{t} & -s\psi^{t} & \varphi^{t}s\psi^{t}+\theta^{t}c\psi^{t}\\ s\psi^{t} & c\psi^{t} & \theta^{t}s\psi^{t}-\varphi^{t}c\psi^{t}\\ -\theta^{t} & \varphi^{t} & 1 \end{array}\right]$$

An ideal body that moves along the track, taking the same orientation as the track frame with a forward velocity *V* and a forward acceleration $\dot{V}$, has the following absolute velocity and acceleration:

(4) $${\bar{\dot{R}}}^{t}=\left[\begin{array}{c} V\\ 0\\ 0 \end{array}\right],\;\;\;\;\;{\bar{\ddot{R}}}^{t}=\left[\begin{array}{c} \dot{V}\\ \rho_{h}V^{2}\\ -\rho_{v}V^{2} \end{array}\right]$$

Similarly, the absolute angular velocity and the absolute angular acceleration of that body are given by:

(5) $${\bar{\omega }}^{t}=\left[\begin{array}{c} \rho_{tw}V\\ \rho_{v}V\\ \rho_{h}V \end{array}\right],\;\;\;\;\;{\bar{\alpha }}^{t}=\left[\begin{array}{c} \rho_{tw}\dot{V}\\ \rho_{v}\dot{V}\\ \rho_{h}\dot{V}+{\rho^{\prime }}_{h}V^{2} \end{array}\right]$$

### 3.3. Kinematics of the Irregular Track

Figure 3 shows the displacement of the rail heads due to irregularity in a cross-section of the track ($Y^{t}-Z^{t}$ plane). The irregularity vectors ${\vec{r}}^{\;lir}$ (lir, *l*eft rail irregularity) and ${\vec{r}}^{\;rir}$ (rir, *r*ight rail irregularity) describe the displacement of the rail centerlines with respect to their design positions. The components of these vectors in the TF are functions of *s*, given by:

(6) $${\bar{r}}^{lir}=\left[\begin{array}{c} 0\\ y^{lir}\\ z^{lir} \end{array}\right],\;\;\;\;\;{\bar{r}}^{rir}=\left[\begin{array}{c} 0\\ y^{rir}\\ z^{rir} \end{array}\right]$$

In the railway industry, the following four combinations of the rail head centerlines irregularities are measured:

Alignment: $\;\;\;\;\;\xi_{al}=(y^{lir}+y^{rir})/2$
Vertical profile: $\;\;\xi_{vp}=(z^{lir}+z^{rir})/2$
Gauge variation: $\;\;\;\;\xi_{gv}=y^{lir}-y^{rir}$
Cross level: $\;\;\;\;\xi_{cl}=z^{lir}-z^{rir}$

The orientation of the rail head frames with respect to the TF is given by the following rotation matrices:

(7) $$\begin{array}{c} A^{t,lrp}=\left[\begin{array}{ccc} 1 & 0 & 0\\ 0 & \cos\left(\beta +\delta \right) & -\sin\left(\beta +\delta \right)\\ 0 & \sin\left(\beta +\delta \right) & \cos\left(\beta +\delta \right) \end{array}\right],\\ A^{t,rrp}=\left[\begin{array}{ccc} 1 & 0 & 0\\ 0 & \cos\left(-\beta +\delta \right) & -\sin\left(-\beta +\delta \right)\\ 0 & \sin\left(-\beta +\delta \right) & \cos\left(-\beta +\delta \right) \end{array}\right], \end{array}$$

where β is the orientation angle of the rail profiles, δ=zlir−zrirzlir−zrir2Lr2Lr is the linearized rotation angle due to the irregularity, and $L_{r}$ is the distance from the track centerline to the rail profile frames. These angles and distance can be observed in Figure 3.

The absolute position vectors of two points, *P* and *Q*, defined in the right and left rail heads, respectively, are given by:

(8) $$\begin{array}{c} R_{P}^{rrp}=R^{t}+A^{t}\left({\bar{r}}^{rrp}+{\bar{r}}^{rir}+A^{t,rrp}{\hat{u}}_{P}^{rrp}\right)\\ R_{Q}^{lrp}=R^{t}+A^{t}\left({\bar{r}}^{lrp}+{\bar{r}}^{lir}+A^{t,lrp}{\hat{u}}_{Q}^{lrp}\right) \end{array}$$

where ${\hat{u}}_{P}^{rrp}$ and ${\hat{u}}_{Q}^{lrp}$ contain the components of the position vector of points *P* and *Q* in the rail head profiles as shown in Figure 3. These vectors are parameterized following the rail head profile geometry:

(9) $${\hat{u}}_{P}^{rrp}=\left[\begin{array}{c} 0\\ s_{2}^{rr}\\ h^{r}\left(s_{2}^{rr}\right) \end{array}\right],\;\;\;\;{\hat{u}}_{Q}^{lrp}=\left[\begin{array}{c} 0\\ s_{2}^{lr}\\ h^{r}\left(s_{2}^{lr}\right) \end{array}\right]$$

where lr and rr stand for “left rail” and “right rail”, and $h^{r}$ is the function that defines the rail head profile.

### 3.4. Kinematics of a Body Moving along the Track

The coordinates used to describe the position and orientation of an arbitrary body *i*, as shown in Figure 2, or the TGMS shown in Figure 1, moving along the track are:

(10) $$q^{i}={\left[\begin{array}{cccccc} s^{i} & r_{y}^{i} & r_{z}^{i} & \varphi^{i} & \theta^{i} & \psi^{i} \end{array}\right]}^{T}$$

where $s^{i}$ is the arc-length along the track of the position of the body, $r_{y}^{i}$ and $r_{z}^{i}$ are the non-zero components of the position vector ${\vec{r}}^{i}$ of the BF with respect to the TF, this is ${\bar{r}}^{i}={\left[\begin{array}{ccc} 0 & r_{y}^{i} & r_{z}^{i} \end{array}\right]}^{T}$, and $\varphi^{i},\;\;\theta^{i}\;\;\mathrm{and}\;\;\psi^{i}$ are three Euler angles (roll, pitch and yaw, respectively) that define the orientation of the BF with respect to the TF. Note that, out of the six coordinates used for the kinematic description of a body, only the arc-length coordinate $s^{i}$ is an absolute coordinate, being the other 5 track-relative coordinates.

The three Euler angles are assumed to be small, such that the following kinematic linearization is used:

(11) $$A^{t,i}≃\left[\begin{array}{ccc} 1 & -\psi^{i} & \theta^{i}\\ \psi^{i} & 1 & -\varphi^{i}\\ -\theta^{i} & \varphi^{i} & 1 \end{array}\right]$$

The absolute position vector of point *P* that belongs to body *i*, as shown in Figure 2, is given by:

(12) $$R_{P}^{i}=R^{t}+A^{t}\left({\bar{r}}^{i}+A^{t,i}{\hat{u}}_{P}^{i}\right)$$

Using basic rigid body kinematics (see details in [17]), the absolute velocity and acceleration of point *P* are given by:

(13) $${\bar{\dot{R}}}_{P}^{i}={\bar{\dot{R}}}^{t}+{\dot{\bar{r}}}^{i}+{\tilde{\bar{\omega }}}^{t}{\bar{r}}^{i}+A^{t,i}\left({\tilde{\hat{\omega }}}^{i}{\hat{u}}_{P}^{i}\right)$$

(14) $${\bar{\ddot{R}}}_{P}^{i}={\bar{\ddot{R}}}^{t}+{\ddot{\bar{r}}}^{i}+\left({\tilde{\bar{\alpha }}}^{t}+{\tilde{\bar{\omega }}}^{t}{\tilde{\bar{\omega }}}^{t}\right){\bar{r}}^{i}+2{\tilde{\bar{\omega }}}^{t}{\dot{\bar{r}}}^{i}+A^{t,i}\left({\tilde{\hat{\alpha }}}^{i}+{\tilde{\hat{\omega }}}^{i}{\tilde{\hat{\omega }}}^{i}\right){\hat{u}}_{P}^{i},$$

where these vectors are projected to the track frame. In order to compute Equations (13) and (14), the orientation matrices, angular velocities and angular accelerations of the different frames need to be computed as a function of the generalized coordinates and velocities. The angular velocity ${\bar{\omega }}^{t}$ and acceleration ${\bar{\alpha }}^{t}$ vectors of the TF are given in Equation (5). The absolute angular velocity of body *i* is obtained as:

(15) $${\hat{\omega }}^{i}={\hat{\omega }}^{t}+{\hat{\omega }}^{t,i}={\left(A^{t,i}\right)}^{T}{\bar{\omega }}^{t}+{\hat{\omega }}^{t,i}$$

The relative angular velocity of body *i* with respect to the TF, under the small-angles assumption, is given by:

(16) $${\hat{\omega }}^{t,i}=\left[\begin{array}{c} {\dot{\varphi }}^{i}\\ {\dot{\theta }}^{i}\\ {\dot{\psi }}^{i} \end{array}\right],\;\;\;\;\;\;{\bar{\omega }}^{t,i}=A^{t,i}{\hat{\omega }}^{t,i}=\left[\begin{array}{ccc} 1 & \psi^{i} & -\theta^{i}\\ -\psi^{i} & 1 & \varphi^{i}\\ \theta^{i} & -\varphi^{i} & 1 \end{array}\right]\left[\begin{array}{c} {\dot{\varphi }}^{i}\\ {\dot{\theta }}^{i}\\ {\dot{\psi }}^{i} \end{array}\right]$$

The absolute angular acceleration of body *i*, ${\hat{\alpha }}^{t}$ is simply calculated as the time-derivative of Equation (15).

## 4. Kinematics of the Computer Vision

**Figure 4:**
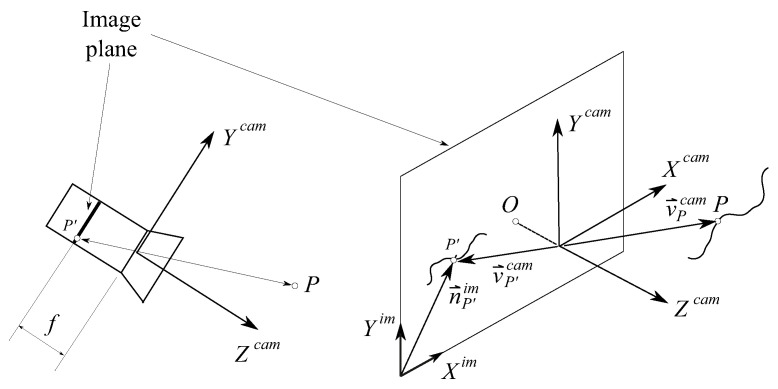
Kinematics of the computer vision.

Using the pin-hole model of the camera, Figure 4 shows the relation between the position vector ${\vec{n}}_{P^{\prime }}^{\;im}$ of an arbitrary point *P* in the camera frame $<X^{cam},Y^{cam},Z^{cam}>$ (in our problem it can be left cam lcam or right cam rcam) and the position vector of the recorded point *P’* in the image plane $<X^{im},Y^{im}>$.

**Figure 5:**
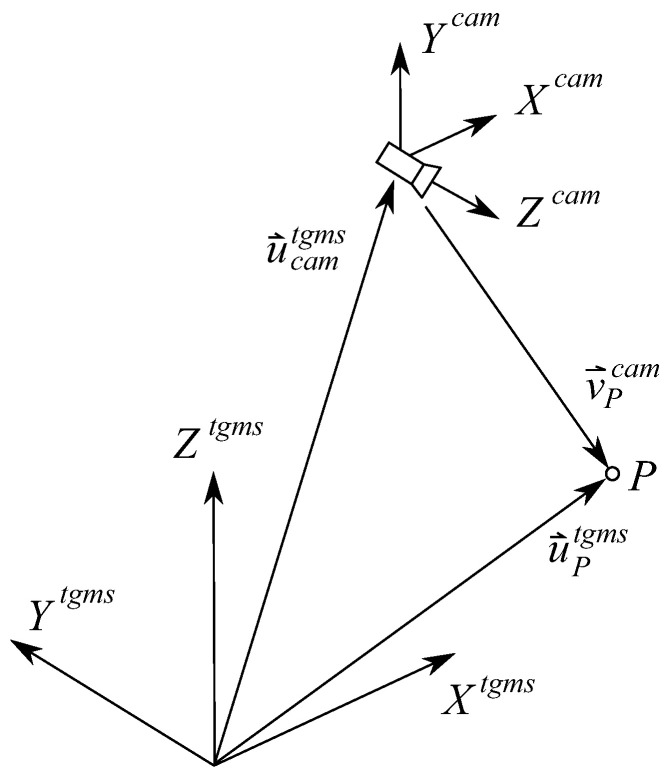
TGMS frame and camera frame.

Figure 5 shows the location of the camera in the TGMS and the relation between the position vector ${\vec{v}}_{P}^{\;cam}$ of the arbitrary point *P* in the camera frame and its position vector ${\vec{u}}_{P}^{\;tgms}$ in the TGMS frame.

The components of vectors ${\vec{n}}_{P^{\prime }}^{\;im}$ and ${\vec{u}}_{P}^{\;tgms}$ are related through the equation [18,19]:

(17) $$c\left[\begin{array}{c} n_{P^{\prime }}^{im}\\ 1 \end{array}\right]=M^{int}M^{ext}\left[\begin{array}{c} {\hat{u}}_{P}^{tgms}\\ 1 \end{array}\right]$$

where *c* is an unknown scalefactor. The matrix product on the right-hand side of this equation is called projection matrix $P=M^{int}M^{ext}$. The column matrix $n_{P^{\prime }}^{im}$ is 2×1 (image is planar), and its components are given in pixel units (dimensionless) while the column matrix ${\hat{u}}_{P}^{tgms}$ is 3×1, and its components are given in meters. Due to these dimensions, the projection matrix **P** is 3×4. Matrix $M^{int}$ is 3×3 and it is called matrix of intrinsic parameters of the camera, and matrix $M^{ext}$ is 3×4, it is called matrix of extrinsic parameters of the camera, and it is given by:

(18) $$M^{ext}=\left[\begin{array}{cc} {\left(A^{tgms,cam}\right)}^{T} & -{\left(A^{tgms,cam}\right)}^{T}{\hat{u}}_{cam}^{tgms} \end{array}\right]$$

Matrices of intrinsic and extrinsic parameters can be experimentally obtained, for example, using the Zhang calibration method [18]. In this investigation, the calibration method is based on Zhang method but adapted to the application at hand. This method is fully detailed in [19].

Just using the 3 scalar equations in Equation (17), the position vector ${\hat{u}}_{P}^{tgms}$ of the point *P* cannot be obtained using the values of $n_{P^{\prime }}^{im}$ because there are 4 unknowns (three components of ${\hat{u}}_{P}^{tgms}$ and the scale factor *c*). One exception occurs when the point *P* moves on a surface whose equation is known in the TGMS frame. This is the case at hand if *P* belongs to the plane highlighted by the laser projector. In this case, the following system of equations can be solved to find ${\hat{u}}_{P}^{tgms}$:

(19) $$\left\{\begin{array}{c} c\left[\begin{array}{c} n_{P^{\prime }}^{im}\\ 1 \end{array}\right]=M^{int}M^{ext}\left[\begin{array}{c} {\hat{u}}_{P}^{tgms}\\ 1 \end{array}\right]\\ A^{las}{\left[u_{P}^{tgms}\right]}_{x}+B^{las}{\left[u_{P}^{tgms}\right]}_{y}+C^{las}{\left[u_{P}^{tgms}\right]}_{z}+D^{las}=0 \end{array}\right.\;$$

where $A^{las},\;\;B^{las},\;\;C^{las}$ and $D^{las}$ are the constants that define the laser plane (left laser, llas, or right laser, rlas, in our case), and ${\left[u_{P}^{tgms}\right]}_{k}\;\;\;k=x,y,z$ means the component *k* of the vector ${\vec{u}}_{P}^{\;tgms}$ in the TGMS frame. The constants that define the laser planes have to be experimentally obtained in the TGMS computer vision-calibration process [19]. Equation (19) is a system of 4 algebraic equations with 4 unknowns that can be used to find vector components ${\hat{u}}_{P}^{tgms}$ using as input data the vector components $n_{P^{\prime }}^{im}$ and the parameters of the cameras and the lasers.

## 5. Detecting the Rail Cross-Section from a Camera Frame

As a result of the solution of Equation (19) for all highlighted pixels in the image frames, a cloud of points $P_{i}$ in the right rail and a cloud of points $Q_{i}$ in the left rail, with position vectors ${\hat{u}}_{P}^{tgms}$ and ${\hat{u}}_{Q}^{tgms}$, respectively, that belong to the rails cross-sections can be identified. In fact, the points do not really belong to cross-sections, just to sections, because the laser planes are not necessarily perpendicular to the rails center line. However, because the relative angles of the TGMS with respect to the TF are very small, the irregularities are also small, and the lasers are set to project the light plane at right angles with respect to the rails, it will be assumed in this paper that the highlighted sections are actually cross-sections.

**Figure 6:**
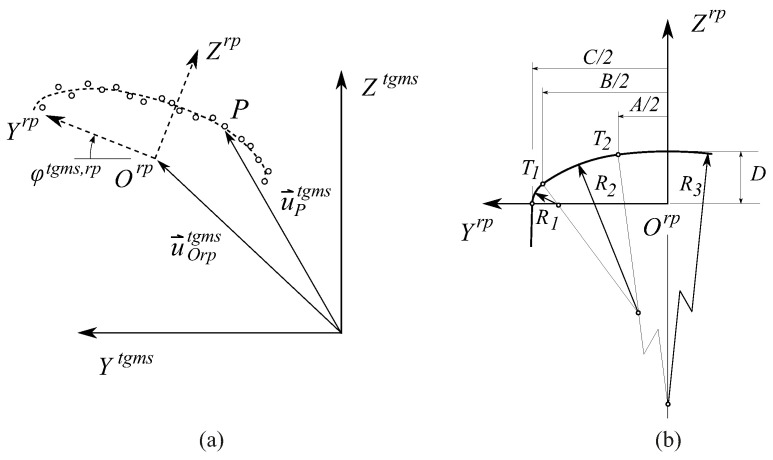
Cloud of points detected with computer vision (**a**). UIC 54 E1 rail-head profile (**b**).

Figure 6a shows a sketch of the cloud of points and, in dashed line, the theoretical rail-head profile. The theoretical rail-head profiles, when they are new, not worn, have a known geometry that is made of circular and straight segments. An example is the UIC 54 E1 rail-head profile shown in Figure 6b. Detecting the rail cross-section from a camera frame can be solved with the well-developed computer vision algorithms of feature detection or feature tracking [20]. However, in this investigation, because the feature to be detected can be represented analytically, an optimization approach has been developed. The optimization problem consists of finding the position ${\hat{u}}_{Orp}^{tgms}$ and angle $\varphi^{tgms,rp}$ of the rail profile that better fits the cloud of points. This will be the assumed position and orientation of the rail head profile (lrp or rrp) in the TGMS frame when the vehicle is moving. The objective function to minimize is the sum of the squares of the distance of the points in the cloud to the theoretical profile curve shown in Figure 6b.

## 6. Equations for Geometry Measurement

**Figure 7:**
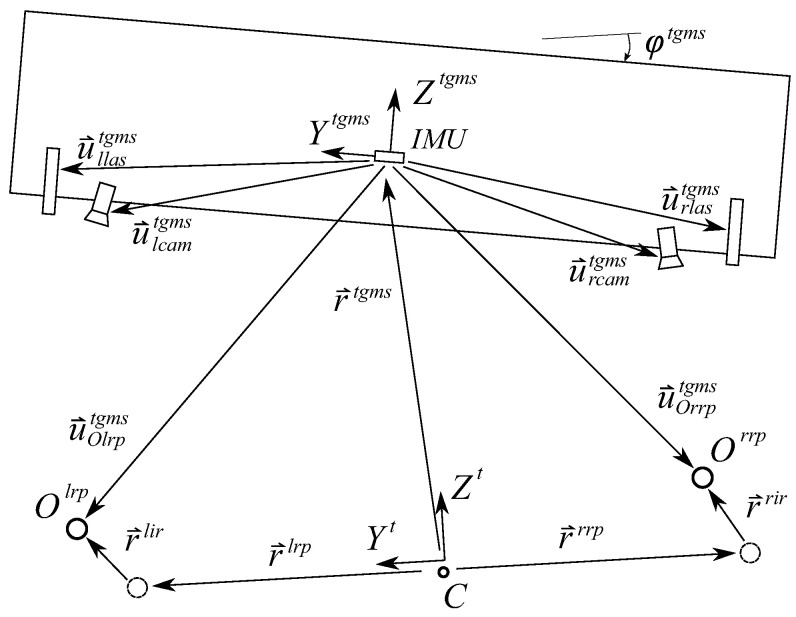
Planar view of the TGMS.

The equations that can be used to measure the track irregularities are easily deduced with the help of Figure 7. In this figure, vectors ${\hat{u}}_{Olrp}^{tgms}\;\;\mathrm{and}\;\;{\hat{u}}_{Orrp}^{tgms}$ are output data of the computer vision algorithm explained in previous section. The following equalities can be easily identified with the help of the figure:

(20) $$\begin{array}{c} {\vec{r}}^{\;tgms}+{\vec{u}}_{Olrp}^{\;tgms}={\vec{r}}^{\;lrp}+{\vec{r}}^{\;lir}\\ {\vec{r}}^{\;tgms}+{\vec{u}}_{Orrp}^{\;tgms}={\vec{r}}^{\;rrp}+{\vec{r}}^{\;rir} \end{array}$$

Subtracting these vector equations, one gets:

(21) $${\vec{u}}_{Olrp}^{\;tgms}-{\vec{u}}_{Orrp}^{\;tgms}={\vec{r}}^{\;lrp}+{\vec{r}}^{\;lir}-\left({\vec{r}}^{\;rrp}+{\vec{r}}^{\;rir}\right)$$

In this equation, the position vector ${\vec{r}}^{tgms}$ of the TGMS does not appear. This vector equation can be projected in the TF, as follows:

(22) $$A^{t,tgms}\left({\hat{u}}_{Olrp}^{tgms}-{\hat{u}}_{Orrp}^{tgms}\right)={\bar{r}}^{lrp}-{\bar{r}}^{rrp}+{\bar{r}}^{lir}-{\bar{r}}^{rir}$$

Using again the small-angles assumption, the Y,Z components of this equation are given by:

(23) $$\left[\begin{array}{cc} 1 & -\varphi^{tgms}\\ \varphi^{tgms} & 1 \end{array}\right]\left[\begin{array}{c} {\left[{\hat{u}}_{Olrp}^{tgms}\right]}_{y}-{\left[{\hat{u}}_{Orrp}^{tgms}\right]}_{y}\\ {\left[{\hat{u}}_{Olrp}^{tgms}\right]}_{z}-{\left[{\hat{u}}_{Orrp}^{tgms}\right]}_{z} \end{array}\right]=\left[\begin{array}{c} 2L^{r}\\ 0 \end{array}\right]+\left[\begin{array}{c} r_{y}^{lir}-r_{y}^{rir}\\ r_{z}^{lir}-r_{z}^{rir} \end{array}\right]$$

where $L^{r}$ is half the distance between the rail-head profiles without irregularities. In this equation, the result ${\bar{r}}^{lrp}-{\bar{r}}^{rrp}={\left[\begin{array}{cc} 2L^{r} & 0 \end{array}\right]}^{T}$ has been used. According to the definition given in Section 3.3, the components of the last column matrix of Equation (23) are the gauge variation (gv) and the cross-level (cl). Therefore, rearranging Equation (23) yields:

(24) $$\begin{array}{c} \xi_{gv}=\left({\left[{\hat{u}}_{Olrp}^{tgms}\right]}_{y}-{\left[{\hat{u}}_{Orrp}^{tgms}\right]}_{y}\right)-\varphi^{tgms}\left({\left[{\hat{u}}_{Olrp}^{tgms}\right]}_{z}-{\left[{\hat{u}}_{Orrp}^{tgms}\right]}_{z}\right)-2L^{r}\\ \xi_{cl}=\varphi^{tgms}\left({\left[{\hat{u}}_{Olrp}^{tgms}\right]}_{y}-{\left[{\hat{u}}_{Orrp}^{tgms}\right]}_{y}\right)+\left({\left[{\hat{u}}_{Olrp}^{tgms}\right]}_{z}-{\left[{\hat{u}}_{Orrp}^{tgms}\right]}_{z}\right) \end{array}$$

Adding the vector equations Equation (20), one gets:

(25) $$2{\vec{r}}^{\;tgms}+{\vec{u}}_{Q}^{\;tgms}+{\vec{u}}_{P}^{\;tgms}={\vec{r}}^{\;lir}+{\vec{r}}^{\;rir}$$

where the fact that ${\vec{r}}^{\;lrp}+{\vec{r}}^{\;rrp}=\vec{0}$ has been used. Using again the small-angles assumption, the Y,Z components of this equation are given by:

(26) $$2\left[\begin{array}{c} r_{y}^{tgms}\\ r_{z}^{tgms} \end{array}\right]+\left[\begin{array}{cc} 1 & -\varphi^{tgms}\\ \varphi^{tgms} & 1 \end{array}\right]\left[\begin{array}{c} {\left[{\hat{u}}_{Olrp}^{tgms}\right]}_{y}+{\left[{\hat{u}}_{Orrp}^{tgms}\right]}_{y}\\ {\left[{\hat{u}}_{Olrp}^{tgms}\right]}_{z}+{\left[{\hat{u}}_{Orrp}^{tgms}\right]}_{z} \end{array}\right]=\left[\begin{array}{c} r_{y}^{lir}+r_{y}^{rir}\\ r_{z}^{lir}+r_{z}^{rir} \end{array}\right]$$

According to the definition given in Section 3.3, the components of the last column matrix of Equation (26) are twice the alignment irregularity ($\xi_{al}$) and twice the vertical profile ($\xi_{vp}$). Therefore, rearranging Equation (26) yields:

(27) $$\begin{array}{c} \xi_{al}=\frac{1}{2}\left({\left[{\hat{u}}_{Olrp}^{tgms}\right]}_{y}+{\left[{\hat{u}}_{Orrp}^{tgms}\right]}_{y}\right)-\frac{\varphi^{tgms}}{2}\left({\left[{\hat{u}}_{Olrp}^{tgms}\right]}_{z}+{\left[{\hat{u}}_{Orrp}^{tgms}\right]}_{z}\right)+r_{y}^{tgms}\\ \xi_{vp}=\frac{\varphi^{tgms}}{2}\left({\left[{\hat{u}}_{Olrp}^{tgms}\right]}_{y}+{\left[{\hat{u}}_{Orrp}^{tgms}\right]}_{y}\right)+\frac{1}{2}\left({\left[{\hat{u}}_{Olrp}^{tgms}\right]}_{z}+{\left[{\hat{u}}_{Orrp}^{tgms}\right]}_{z}\right)+r_{z}^{tgms} \end{array}$$

Therefore, Equations (24) and (27) can be used to find all track irregularities. The following conclusions are highlighted:

(1) The calculation of the relative track irregularities ($\xi_{gv}$ and $\xi_{cl}$), as shown in Equation (24), needs as an input the output of the computer vision ${\hat{u}}_{Olrp}^{tgms}\;\;\mathrm{and}\;\;{\hat{u}}_{Orrp}^{tgms}$ and the roll angle of the TGMS with respect to the track $\varphi^{tgms}$.
(2) The calculation of the absolute track irregularities ($\xi_{al}$ and $\xi_{vp}$), as shown in Equation (27), needs, in addition, the relative trajectory ${\bar{r}}^{tgms}$ of the TGMS with respect to the TF.

The next section is devoted to the calculation of the TGMS to TF relative orientation, that includes the calculation of $\varphi^{tgms}$. Section 9 is devoted to the calculation of the relative trajectory ${\bar{r}}^{tgms}$ of the TGMS with respect to the TF.

## 7. Measurement of TGMS to TF Relative Orientation

The calculation of the relative rotation of the TGMS with respect to the TF requires the calculation of the absolute orientation of the TGMS and the absolute orientation of the TF. As given in Equations (2) and (3), finding the orientation of the TF just requires the instantaneous value of the arc-length *s*, because the track design geometry is known. The value of *s* has to be known accurately. The odometry algorithm explained in the next section has been developed to this end.

The calculation of the absolute orientation of the TGMS is obtained with the IMU sensor. Most IMU sensors come with their own internal algorithm to calculate the orientation angles or quaternions. However, these algorithms are not accurate in the application at hand. They are based on the sensor fusion of the data of the gyroscope, the accelerometer and the magnetometer, as follows:

1. The gyroscope provides three signals that are proportional to the components of the angular velocity vector in the sensor frame (the sensor frame is assumed to be parallel to the TGMS frame), as follows:
  (28) $$\omega^{imu}={\hat{\omega }}_{abs}^{tgms}$$
  These components are non-linearly related to the coordinates that define the TGMS orientation and their time-derivatives, as shown later.
2. The accelerometer signals are proportional to the components of the absolute acceleration in the local frame plus the absolute gravity vector field, as follows:
  (29) $$a^{imu}={\hat{\ddot{R}}}^{tgms}+{\left(A^{tgms}\right)}^{T}{\left[\begin{array}{ccc} 0 & 0 & g \end{array}\right]}^{T}$$
  This is the absolute acceleration in the sensor frame, plus the gravitational constant *g*, that is assumed to act in the absolute *Z* direction.
3. The magnetometer signals are proportional to the components of the Earth’s magnetic field in the local frame. This information can be used to find the direction of the Earth’s magnetic north.

In the algorithm developed in this investigation to find the TGMS absolute orientation, the magnetometer’s signals are not used. This is mainly because out of the three Euler angles needed to define the orientation, the yaw angle lacks interest in our application. It is mainly the yaw angle which can be identified with the help of the magnetometer.

Following an Euler’s angle sequence yaw-pitch-roll, as done with the TF to get the rotation matrix given in Equation (2), the absolute angular velocity of the TGMS that appear in Equation (28) can be obtained as follows:

(30) $$\begin{array}{c} {\hat{\omega }}_{abs}^{tgms}=\left[\begin{array}{c} -\sin\theta_{abs}^{tgms}\\ \cos\theta_{abs}^{tgms}\sin\varphi_{abs}^{tgms}\\ \cos\theta_{abs}^{tgms}\cos\varphi_{abs}^{tgms} \end{array}\right]{\dot{\psi }}_{abs}^{tgms}+\left[\begin{array}{c} 0\\ \cos\varphi_{abs}^{tgms}\\ -\sin\varphi_{abs}^{tgms} \end{array}\right]{\dot{\theta }}_{abs}^{tgms}+\left[\begin{array}{c} 1\\ 0\\ 0 \end{array}\right]{\dot{\varphi }}_{abs}^{tgms}=\\ =\left[\begin{array}{ccc} 1 & 0 & -\sin\theta_{abs}^{tgms}\\ 0 & \cos\varphi_{abs}^{tgms} & \cos\theta_{abs}^{tgms}\sin\varphi_{abs}^{tgms}\\ 0 & -\sin\varphi_{abs}^{tgms} & \cos\theta_{abs}^{tgms}\cos\varphi_{abs}^{tgms} \end{array}\right]\left[\begin{array}{c} {\dot{\varphi }}_{abs}^{tgms}\\ {\dot{\theta }}_{abs}^{tgms}\\ {\dot{\psi }}_{abs}^{tgms} \end{array}\right]={\hat{G}}^{tgms}{\dot{\Phi }}_{abs}^{tgms}, \end{array}$$

where $\Phi_{abs}^{tgms}={\left[\begin{array}{ccc} \varphi_{abs}^{tgms} & \theta_{abs}^{tgms} & \psi_{abs}^{tgms} \end{array}\right]}^{T}$ is the set of absolute angles (with respect to the global frame) of the TGMS. This expression is non-linear in terms of the TGMS absolute angles, but linear in term of their time-derivative. This equation can be inverted to isolate the time-derivatives of the angles, as follows:

(31) $$\left[\begin{array}{c} \dot{\varphi }\\ \dot{\theta }\\ \dot{\psi } \end{array}\right]={\left(\hat{G}\right)}^{-1}\hat{\omega }=\left[\begin{array}{ccc} 1 & \frac{\sin\varphi \sin\theta }{\cos\theta } & \frac{\cos\varphi \sin\theta }{\cos\theta }\\ 0 & \cos\varphi & -\sin\varphi \\ 0 & \frac{\sin\varphi }{\cos\theta } & \frac{\cos\varphi }{\cos\theta } \end{array}\right]\left[\begin{array}{c} {\hat{\omega }}_{x}\\ {\hat{\omega }}_{y}\\ {\hat{\omega }}_{z} \end{array}\right]$$

where, as done in the rest of this section, the superscript tgms and subscript abs have been eliminated in the symbols for simplicity. Using again the small-angles assumption of the roll and pitch angles, the following linearized formulas result:

(32) $$\begin{array}{c} \dot{\varphi }={\hat{\omega }}_{x}+\frac{\sin\varphi \sin\theta }{\cos\theta }{\hat{\omega }}_{y}+\frac{\cos\varphi \sin\theta }{\cos\theta }{\hat{\omega }}_{z}≃{\hat{\omega }}_{x}+\theta {\hat{\omega }}_{z}\\ \dot{\theta }=\cos\varphi {\hat{\omega }}_{y}-\sin\varphi {\hat{\omega }}_{z}≃{\hat{\omega }}_{y}-\varphi {\hat{\omega }}_{z}\\ \dot{\psi }=\frac{\sin\varphi }{\cos\theta }{\hat{\omega }}_{y}+\frac{\cos\varphi }{\cos\theta }{\hat{\omega }}_{z}≃\varphi {\hat{\omega }}_{y}+{\hat{\omega }}_{z} \end{array}$$

For the accelerometer signals, Equation (29) can be linearized as follows:

(33) $$a^{imu}≃{\hat{\ddot{R}}}^{\mathrm{tgms}}+g\left[\begin{array}{c} -\theta \\ \varphi \\ 1 \end{array}\right]$$

where:

(34) $${\hat{\ddot{R}}}^{\mathrm{tgms}}={\left[A^{t,\mathrm{tgms}}\right]}^{T}{\bar{\ddot{R}}}^{\mathrm{tgms}},\;\;\;\;\;\;\;A^{t,\mathrm{tgms}}≃\left[\begin{array}{ccc} 1 & -\psi^{tgms} & \theta^{tgms}\\ \psi^{tgms} & 1 & -\varphi^{tgms}\\ -\theta^{tgms} & \varphi^{tgms} & 1 \end{array}\right]$$

Again, because of the small-angles assumption and for the sake of simplicity, the following approximation is used:

(35) $${\hat{\ddot{R}}}^{\mathrm{tgms}}≃{\bar{\ddot{R}}}^{\mathrm{tgms}}$$

Using the result of Equation (14), the absolute acceleration of the TGMS is given by:

(36) $${\bar{\ddot{R}}}^{\mathrm{tgms}}={\bar{\ddot{R}}}^{t}+{\ddot{\bar{r}}}^{\mathrm{tgms}}+\left({\tilde{\bar{\alpha }}}^{t}+{\tilde{\bar{\omega }}}^{t}{\tilde{\bar{\omega }}}^{t}\right){\bar{r}}^{\mathrm{tgms}}+2{\tilde{\bar{\omega }}}^{t}{\dot{\bar{r}}}^{\mathrm{tgms}}$$

**Figure 8:**
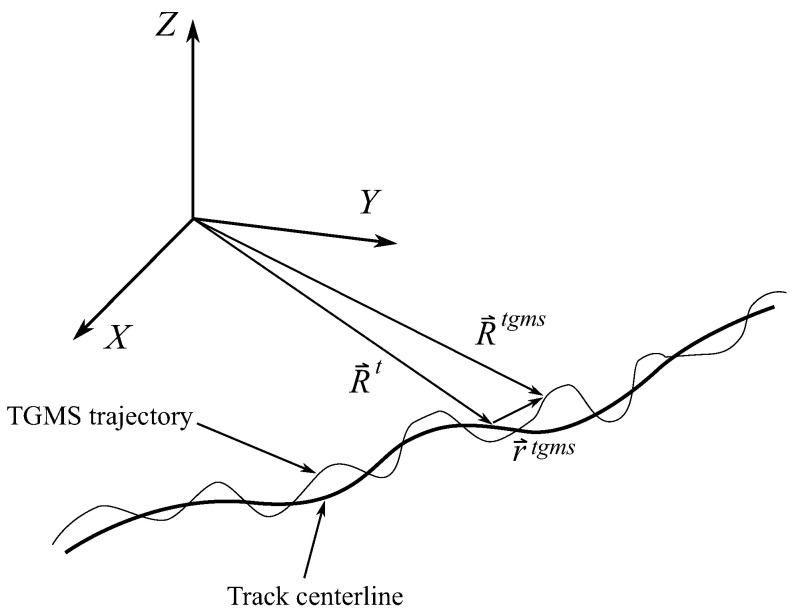
TGMS trajectory.

In this equation, the last three terms on the right-hand side are unknown. These are the TGMS to TF relative acceleration, the tangential and centripetal relative accelerations and the Coriolis acceleration, respectively. In order to calculate these terms, the value of the relative position vector ${\bar{r}}^{\mathrm{tgms}}$, velocity ${\dot{\bar{r}}}^{\mathrm{tgms}}$ and acceleration ${\ddot{\bar{r}}}^{\mathrm{tgms}}$ of the TGMS with respect to the TF are needed. As it can be observed in Figure 8, the trajectory followed by the TGMS when the vehicle is moving is a 3D curve that slightly differs with respect to the track centerline. In fact, the difference between these 3D curves is needed to measure the absolute track irregularities. However, in order to find the TGMS to TF relative orientation, it is going to be assumed that the acceleration equals the one of a particle moving along the track centerline, this is:

(37) $${\bar{\ddot{R}}}^{\mathrm{tgms}}≃{\bar{\ddot{R}}}^{t}=\left[\begin{array}{c} \dot{V}\\ \rho_{h}V^{2}\\ -\rho_{v}V^{2} \end{array}\right]$$

Therefore, the influence of the last three terms in Equation (36) has been neglected.

The sequence of approximations that has been assumed to calculate the acceleration of the TGMS may look rough for some readers. However, it has to be taken into account that in most algorithms used to find orientation from IMU’s signals [21,22] the true acceleration measured by the sensor is neglected in comparison with the gravity term in Equation (33). In the application at hand, this approximation is not accurate and the “compensation” introduced with Equation (36) is much better than nothing.

Finally, the following measurement equation is used to account for the accelerometer signals:

(38) $$a_{corr}^{imu}=a_{filt}^{imu}-\left[\begin{array}{c} \dot{V}\\ \rho_{h}V^{2}\\ -\rho_{v}V^{2} \end{array}\right]\approx g\left[\begin{array}{c} -\theta \\ \varphi \\ 1 \end{array}\right]$$

where $a_{corr}^{imu}$ is the “corrected” accelerometer signal and $a_{filt}^{imu}$ is the low-pass filtered acceleration signal. The reason for low-pass filtering the acceleration signals is that the effect of the TGMS to TF relative accelerations that have been neglected in Equation (36) contribute to relatively high-frequencies to the signals. This way their contribution is, in part, filtered out of the signals.

Using the two first gyroscope equations given in Equation (30) and the last two accelerometer equations given in Equation (38), the following linear dynamic system can be formulated in state-space form:

(39) $$\begin{array}{c} \dot{x}=\left[\begin{array}{c} \dot{\varphi }\\ \dot{\theta } \end{array}\right]=\left[\begin{array}{cc} 0 & {\hat{\omega }}_{z}\\ -{\hat{\omega }}_{z} & 0 \end{array}\right]\left[\begin{array}{c} \varphi \\ \theta \end{array}\right]+\left[\begin{array}{c} {\hat{\omega }}_{x}\\ {\hat{\omega }}_{y} \end{array}\right]=\mathrm{Fx}+u\\ z=\left[\begin{array}{c} {\left(a_{corr}^{imu}\right)}_{x}\\ {\left(a_{corr}^{imu}\right)}_{y} \end{array}\right]=\left[\begin{array}{cc} 0 & -g\\ g & 0 \end{array}\right]\left[\begin{array}{c} \varphi \\ \theta \end{array}\right]=\mathrm{Hx} \end{array}$$

where the state vector $x={\left[\begin{array}{cc} \varphi & \theta \end{array}\right]}^{T}$ just includes the roll and pitch angles, the state transition matrix F is not constant but depends on the measured vertical angular velocity, the measurement matrix H is constant, the measurement vector z includes the *x* and *y* measured-corrected accelerations and the input vector u includes the *x* and *y* measured angular velocities. The linear system shown in (39) is a very simple set of equations that can be used with a standard Kalman filter algorithm to find the TGMS to TF relative angles φ and θ.

## 8. Odometry Algorithm

The odometry algorithm presented here can be used when the TGMS has no access to the data of a precise odometer of the vehicle and/or a GNSS cannot be used, for example, as it happens in underground trains. Underground trains use to be metropolitan. Being metropolitan, there used to be many narrow curved sections. Curved sections facilitate the method presented next.

**Figure 9:**
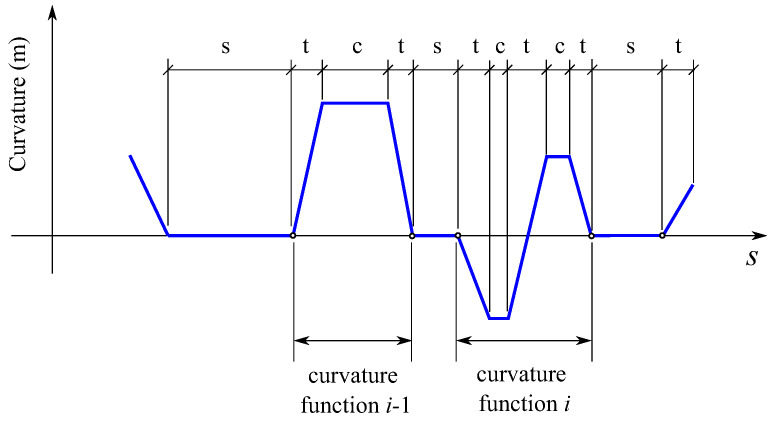
Design horizontal curvature of a railway track.

As shown in Figure 9, the design geometry of a railway track (horizontal profile, as explained in Section 3.2) is a succession of segments of three types: straight (*s* in the figure) with zero curvature, circular (*c* in the figure) with constant curvature and transitions (*t* in the figure) with linearly varying curvature. The curvature function can be decomposed into a set of zero segments (straight segments) plus a set of curvature functions that have trapezoidal shape (normal curve) or double-trapezoidal shape (*S-curve*). The location of the curvature functions (start and end points) is exactly identified along the track using the design geometry provided by the track preprocessor.

The curvature of the track can be experimentally approximated in the TGMS with the installed sensors. The curvature of the trajectory followed by the TGMS can be obtained as:

(40) $$\rho_{h}^{\exp}≃\frac{{\hat{\omega }}_{z}^{tgms}}{V}$$

Of course, this approximate measure is a noisy version of the track horizontal curvature. However, experimental measures show that the overall shape of the curvature functions can be clearly obtained with this approximation.

**Figure 10:**
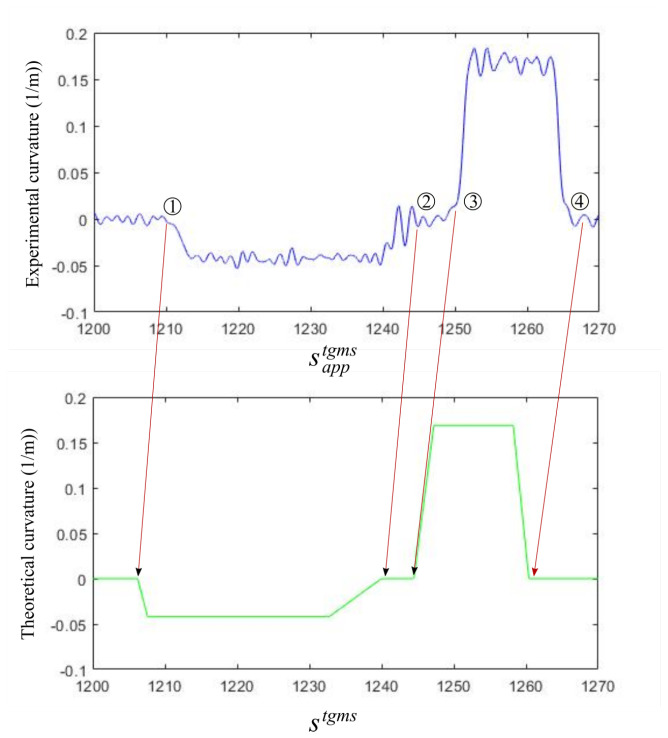
Odometry algorithm.

**Figure 11:**
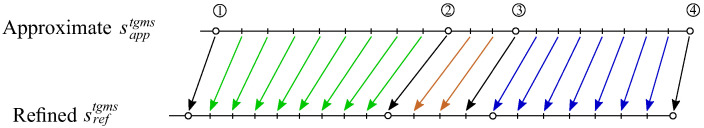
Correction of ${}^{tgms}$.

The concept of the odometry algorithm is to monitor the experimental curvature during the ride of the train using Equation (40) and to store the data together with the approximate coordinate $s_{app}^{tgms}$ obtained with the help of the installed encoder and the assumption of rolling-without-slipping of the wheel. The plot of the experimental curvature looks like the plot at the top of Figure 10. Using the track preprocessor, the design value of the curvature of the track $\rho_{h}^{design}$ in the area where the train is located, may look like the lower plot in Figure 10. As shown in the figure, this information can be used to correct the value of $s_{app}^{tgms}$ at points 1, 2, 3 and 4 located at the entry or exit of the curves. Measures of $s_{app}^{tgms}$ between these corrected points are also corrected using a linear mapping, as shown in Figure 11. Where, black arrows mean odometry corrections due to the algorithm output. Coloured arrows mean odometry correction using linear interpolation of the algorithm output. Different colours are just used to distinguish the curvature function sections

The problem is how to detect the entry and exit of the curves using the functions $\rho_{h}^{\exp}\left(s_{app}^{tgms}\right)$ and $\rho_{h}^{design}\left(s^{tgms}\right)$. In fact, it is the exit of the curves that is detected first. Once the TGMS leaves a curve, the shape of the curvature function that the TGMS has ahead is known. Therefore, when the measured curvature $\rho_{h}^{\exp}\left(s_{app}^{tgms}\right)$ “looks similar” to the expected curvature function $\rho_{h}^{design}\left(s^{tgms}\right)$, the exit of the curve has been reached. This similarity is computed by calculating at each instant the squared-error of the experimentally measured curvature and the expected curvature function, as follows:

(41) $$e2\left(s\right)=\int_{\bar{s}=s-\Delta s}^{\bar{s}=s}{\left[\rho_{h}^{\exp}\left(\bar{s}\right)-\rho_{h}^{design}\left(\bar{s}-s+s_{exit}\right)\right]}^{2}\;d\bar{s}$$

where $e2\left(s\right)$ is the squared error (*s* substitute $s_{app}^{tgms}$ for simplicity in the formula), Δs is the length of the expected curvature function and $s_{exit}$ is the location of the exit of the curve in the design geometry. For a better accuracy, the value of the squared error is normalized for each curvature function using the following factor:

(42) $$I2=\int_{\bar{s}=0}^{\bar{s}=\Delta s}{\left[\rho_{h}^{design}\left(\bar{s}\right)\right]}^{2}\;d\bar{s}$$

The normalization factors, that are different for each curvature function, are of course computed in a preprocesing stage. The normalized squared-error is given by:

(43) $$ne2\left(s\right)=\frac{e2\left(s\right)}{I2}$$

**Figure 12:**
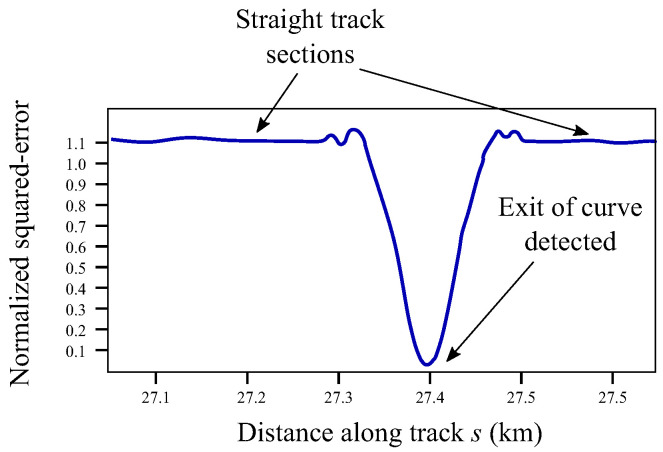
Normalized squared-error.

Thanks to the normalization, the value of ne2 varies between approximately 1, in straight track sections, and 0 when there is a perfect matching between $\rho_{h}^{\exp}\left(s_{app}^{tgms}\right)$ and $\rho_{h}^{design}\left(s^{tgms}\right)$. A typical plot of the function is observed in Figure 12. This function used to be smooth, such that detecting the local minimum that indicates the detection of the exit of the curve is a very easy task. Once the exit of the curve is detected, the expected curvature function is substituted by the next curve ahead along the track. It can be shown that this method can be run in real-time. The main computational cost is the one associated with the calculation of the integral given in Equation (41).

## 9. Measurement of TGMS to TF Relative Motion

The final and most difficult step needed to find the absolute irregularities of the track using Equation (27) is to obtain the relative trajectory ${\bar{r}}^{tgms}$ of the TGMS with respect to the TF shown in Figure 8. Equation (36) can be treated as a second order differential equation in terms of ${\bar{r}}^{tgms}$. This equation has the following scalar components:

(44) $$\begin{array}{c} {\bar{\ddot{R}}}^{tgms}=\left[\begin{array}{c} \dot{V}\\ \rho_{h}V^{2}\\ -\rho_{v}V^{2} \end{array}\right]+\left[\begin{array}{c} 0\\ {\ddot{r}}_{y}^{tgms}\\ {\ddot{r}}_{z}^{tgms} \end{array}\right]+\\ \left[\begin{array}{c} -r_{y}^{tgms}\left(\dot{V}\rho_{h}+V^{2}\left({\rho^{\prime }}_{h}-\rho_{tw}\rho_{v}\right)\right)+r_{z}^{tgms}\left(V^{2}\rho_{tw}\rho_{h}+\dot{V}\rho_{v}\right)\\ -r_{y}^{tgms}\left(V^{2}\left(\rho_{tw}^{2}+\rho_{h}^{2}\right)\right)-r_{z}^{tgms}\left(-V^{2}\rho_{v}\rho_{h}+\dot{V}\rho_{tw}\right)\\ r_{y}^{tgms}\left(V^{2}\rho_{v}\rho_{h}+\dot{V}\rho_{tw}\right)-r_{z}^{tgms}\left(V^{2}\left(\rho_{tw}^{2}+\rho_{v}^{2}\right)\right) \end{array}\right]+\\ +2\left[\begin{array}{c} V{\dot{r}}_{z}^{tgms}\rho_{v}-V{\dot{r}}_{y}^{tgms}\rho_{h}\\ -V{\dot{r}}_{z}^{tgms}\rho_{tw}\\ V{\dot{r}}_{y}^{tgms}\rho_{tw} \end{array}\right] \end{array}$$

Alternatively, the absolute acceleration of the TGMS can be obtained using the measurement of the accelerometer given in Equation (29). If the vectors in Equation (29) are projected to the TF, it yields:

(45) $$\begin{array}{c} {\bar{\ddot{R}}}^{tgms}=A^{t,tgms}a^{imu}-{\left(A^{t}\right)}^{T}{\left[\begin{array}{ccc} 0 & 0 & g \end{array}\right]}^{T}=\\ \left[\begin{array}{c} a_{x}^{imu}-a_{y}^{imu}\psi^{tgms}+a_{z}^{imu}\theta^{tgms}\\ a_{y}^{imu}+a_{x}^{imu}\psi^{tgms}-a_{z}^{imu}\varphi^{tgms}\\ a_{z}^{imu}-a_{x}^{imu}\theta^{tgms}+a_{y}^{imu}\varphi^{tgms} \end{array}\right]+\left[\begin{array}{c} g\theta^{t}\\ -g\varphi^{t}\\ -g \end{array}\right] \end{array}$$

where the small-angles assumption has been used again. The left-hand sides of Equations (44) and (45) represent the same physical magnitudes, therefore, the right hand sides of these equations can be equated. Using just the second and third components of the right hand sides and rearranging yields:

(46) $$\begin{array}{c} \left[\begin{array}{c} {\ddot{r}}_{y}^{tgms}\\ {\ddot{r}}_{z}^{tgms} \end{array}\right]+\left[\begin{array}{cc} 0 & -2V\rho_{tw}\\ 2V\rho_{tw} & 0 \end{array}\right]\left[\begin{array}{c} {\dot{r}}_{y}^{tgms}\\ {\dot{r}}_{z}^{tgms} \end{array}\right]+\\ \left[\begin{array}{cc} -V^{2}\left(\rho_{tw}^{2}+\rho_{h}^{2}\right) & V^{2}\rho_{v}\rho_{h}-\dot{V}\rho_{tw}\\ V^{2}\rho_{v}\rho_{h}+\dot{V}\rho_{tw} & -V^{2}\left(\rho_{tw}^{2}+\rho_{v}^{2}\right) \end{array}\right]\left[\begin{array}{c} r_{y}^{tgms}\\ r_{z}^{tgms} \end{array}\right]=\\ \left[\begin{array}{c} a_{y}^{imu}+a_{x}^{imu}\psi^{tgms}-a_{z}^{imu}\varphi^{tgms}-g\varphi^{t}-\rho_{h}V^{2}\\ a_{z}^{imu}-a_{x}^{imu}\theta^{tgms}+a_{y}^{imu}\varphi^{tgms}-g+\rho_{v}V^{2} \end{array}\right] \end{array}$$

This is a 2nd order linear system of ordinary differential equations (ODE) with time-variant coefficients (linear time-varying system, LTV). This ODE has to be integrated forward in time to find the TGMS to TF relative trajectory ($r_{y}^{tgms}\left(t\right)\;\;\mathrm{and}\;\;r_{z}^{tgms}\left(t\right)$). The inputs of these equations are all calculated or measured so far. These inputs are:

1. The accelerometer data $a^{\mathrm{imu}}$.
2. The instantaneous forward velocity *V* and acceleration $\dot{V}$ of the vehicle. This is obtained from the encoder data.
3. The position $s^{tgms}$ of the TGMS along the track. This is the output of the odometry algorithm explained in previous section. The position $s^{tgms}$ is used as an entry to the track preprocessor to get the track design cant angle $\varphi^{t}$ and the curvatures $\rho_{tw},\;\;\rho_{v}\;\mathrm{and}\;\;\rho_{tw}$.
4. The relative orientation of the TGMS with respect to the TF that is calculated in Section 7.

In the case of a tangent (straight) track, where all track curvatures are zero, Equation (46) reduces to:

(47) $$\left[\begin{array}{c} {\ddot{r}}_{y}^{tgms}\\ {\ddot{r}}_{z}^{tgms} \end{array}\right]=\left[\begin{array}{c} a_{y}^{imu}+a_{x}^{imu}\psi^{tgms}-a_{z}^{imu}\varphi^{tgms}\\ a_{z}^{imu}-a_{x}^{imu}\theta^{tgms}+a_{y}^{imu}\varphi^{tgms}-g \end{array}\right]$$

Numerical integration of Equation (46) can be used to find the TGMS to TF relative motion. However, a close look to Equation (46) shows that these differential equations are equivalent to the equations of motion of a linear 2-degrees of freedom system with negative-definite stiffness and damping matrices. It is a highly unstable system. Therefore, serious problems of drift of the resulting displacements are expected. As a result that the track irregularity is a relatively-high frequency component of the track geometry (being the design geometry the low-frequency component), the drift of the solution can be solved using several methods, like these two:

1. Using a digital signal processing approach for the integration that combines double-integration and high-pass filtering of the signal [3].
2. Using a Kalman filter approach that adds to the system dynamic equations, Equation (46), a set of measurements. These measurements are virtual sensors that in practice always provide a zero value of the TGMS to TF relative motion. The assumed covariance of these measurements is the expected covariance of the track irregularities. This method has been successfully applied in [23] to eliminate the drift in the results while keeping a good accuracy.

The second method is used in this investigation to get the results presented in Section 11.

## 10. Experimental Setup

**Figure 13:**
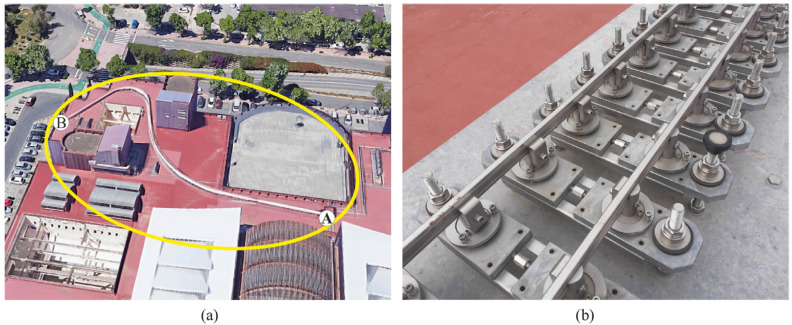
Scaled track at the School of Engineering, University of Seville. (**a**) Aerial view, (**b**) detail of track supports.

A scale track has been built at the rooftop of the School of Engineering at the University of Seville. Figure 13 on the left shows an aerial view of the 90 m-track. The scale is approximately 1:10 (5-inch gauge). The track includes a tangent section (next to end A), a 26 m-curved section and a 12 m-curved section, among other features. The track is supported on a set of mechanisms that can be observed on the right of the figure that are separated 10 cm. These mechanisms can be used to create any irregularity profile. Details of the track design can be found in [24].

**Figure 14:**
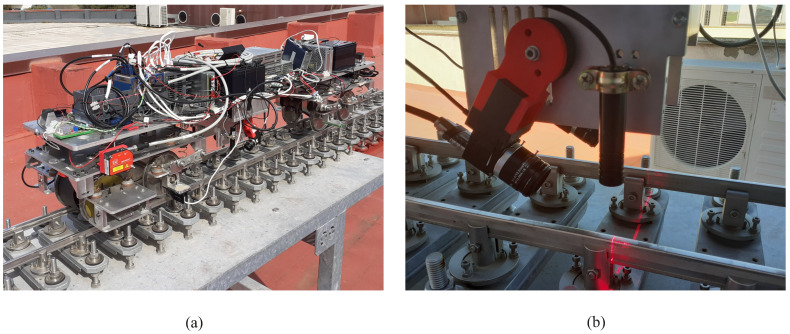
Scale vehicle, (**a**) global view of instrumented scale vehicle, (**b**) detail showing video cameras and laser beam.

Figure 14 shows the scale vehicle that has been used to install the TGMS. The vehicle has a classical architecture of 4 wheelsets, 2 bogies and 1 car-body. The TGMS is attached to the car-body. The right picture shows a detail of the vehicle where the video cameras and the laser beam can be observed. The vehicle drive is an electric motor with a chain transmission. Details of the vehicle design can be found in [24]. The TGMS is equipped with two video cameras Ximea MQ003CG-CM. The VGA cameras have a resolution of 648 × 488 pixels. They include a CMOS RGB bayer matrix sensor. Their maximum frame acquisition rate reaches 500 fps. The camera has a C-mount lens FUJINN 2/3” 12.5 F1.2–F1.6 MI E1.5MP MV that allows the adjustment of focus and exposure. The projection lasers have a power of 10 mW and they illuminate in red. The IMU is a LORD Microstrain 3DM-GX5-25 AHRS, an triaxial accelerometer, gyroscope and magnetometer industrial sensor fully calibrated and temperature compensate. It has a maximum sampling rate of 1 kH. The accelerometer has two different measuring ranges ±8 g (25 g/$\sqrt{\mathrm{Hz}}$) or ±20 g (80 g/$\sqrt{\mathrm{Hz}}$). The gyroscope has an angle random walk of 0.3${}^{\circ }/\sqrt{h}$. The traveled distance by the vehicle along the track is obtained using a precision digital encoder Kubler 2400 series. It is a two channel encoder with up to 1440 pulses per revolution.

**Figure 15:**
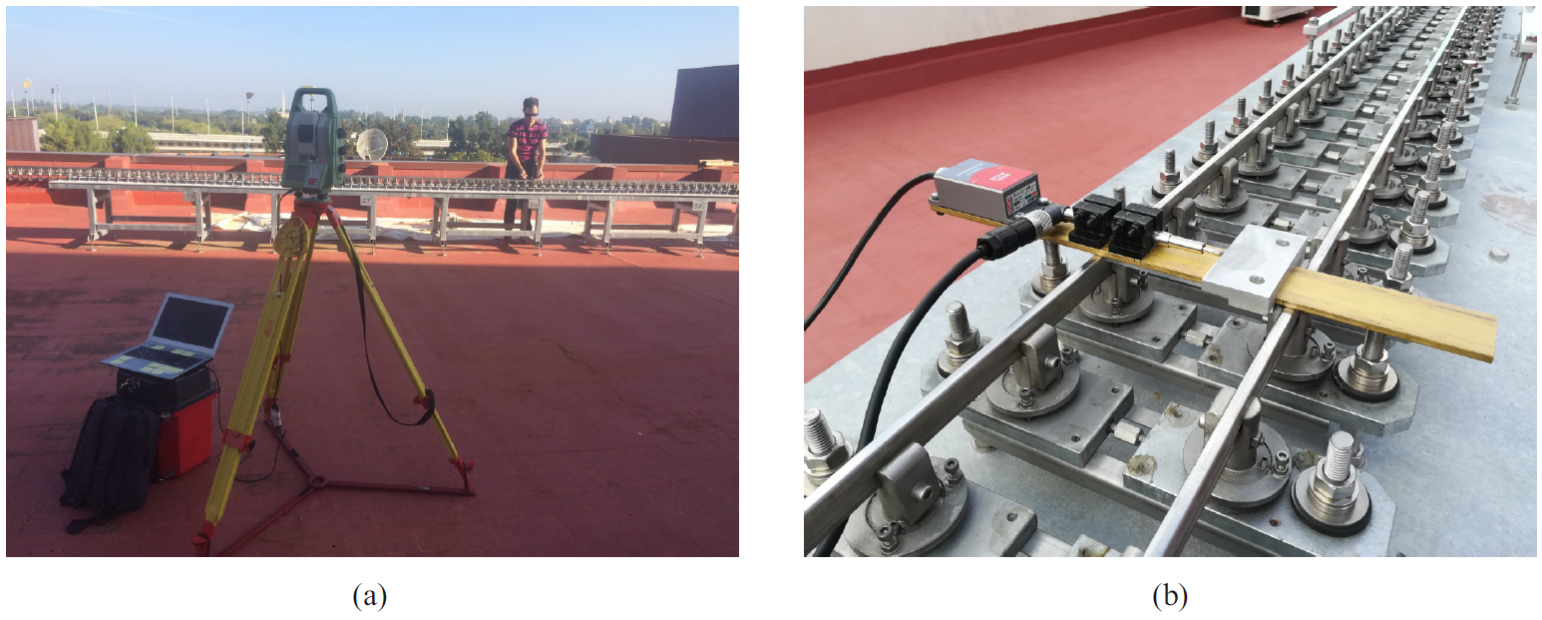
Irregularity measurement. (**a**) Robotic total station, (**b**) LVDT and inclinometer.

In order to have a reference value of the track irregularities, an accurate track geometry measurement has been performed. The relative irregularities have been measured with the instrument that can be observed in the right of Figure 15. This instrument slides along the track. It includes a LVDT to measure the gauge variation and an inclinometer to measure the cross-level. In order to measure the absolute irregularities, alignment and longitudinal profile, the total station shown in the left hand side of the figure has been used. Details of the track geometry measurement can be found in [24].

## 11. Computer Implementation and Comparison of TGMS Measurement with Reference Irregularity

The methods and algorithms described in this paper have been implemented in Matlab environment. However, only built in functions of the basic package have been used, avoiding the use of functions, for example, of the computer vision toolbox. The program that produces the track irregularity out of the sensor data includes the following modules:

1. Pre-process: Camera calibration module. It finds the cameras’ intrinsic and extrinsic parameters using pictures of a checkerboard pattern [19].
2. Pre-process: Track pre-processor module. It is set to provide the design geometry of the track.
3. Process: Computer vision module. It finds the position and orientation of the rail cross-sections using the recorded frames and the method described in Section 5.
4. Process: Odometry module. As described in Section 8, it finds the position, velocity and acceleration of the TGMS every time instant.
5. Process: TGMS orientation module. It finds the orientation of the TGMS using the method described in Section 7.
6. Process: TGMS trajectory module. It finds the relative trajectory of the TGMS with respect to the TF using the method described in Section 9.
7. Process: Irregularity module. It calculates the track irregularities with the method of Section 6.

**Figure 16:**
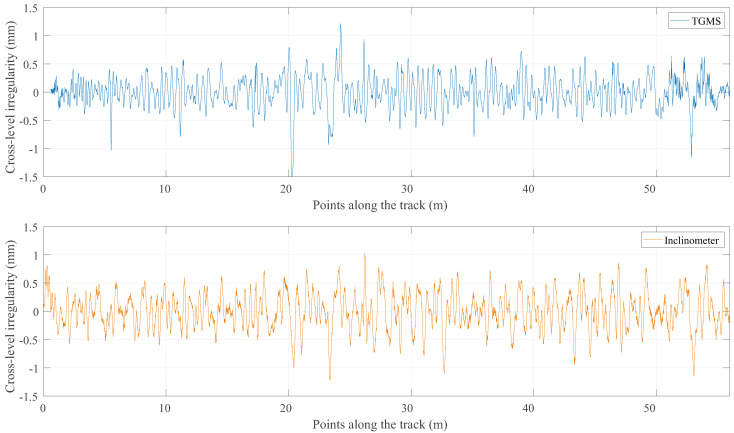
Measurements of cross level.

**Figure 17:**
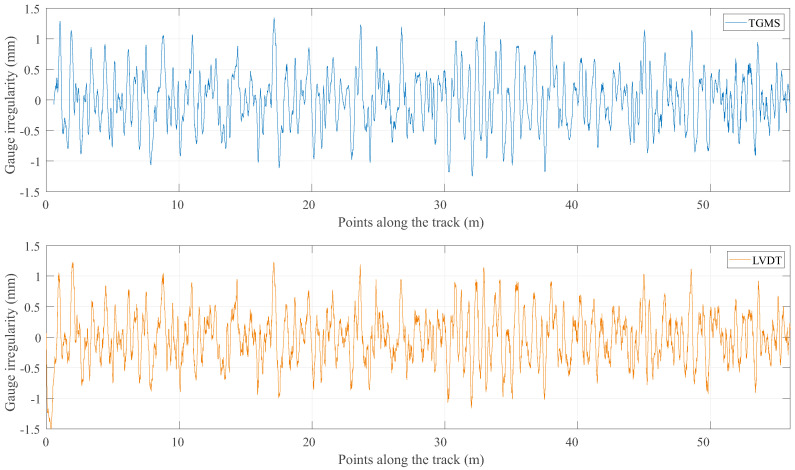
Measurements of gauge variation.

**Figure 18:**
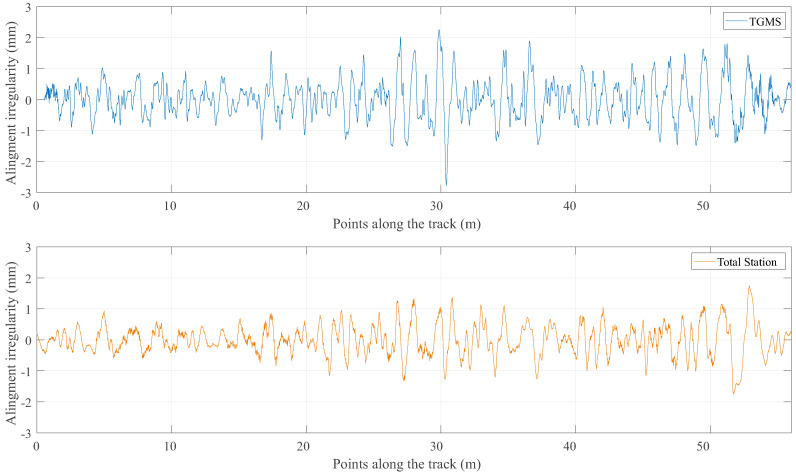
Measurements of alignment.

**Figure 19:**
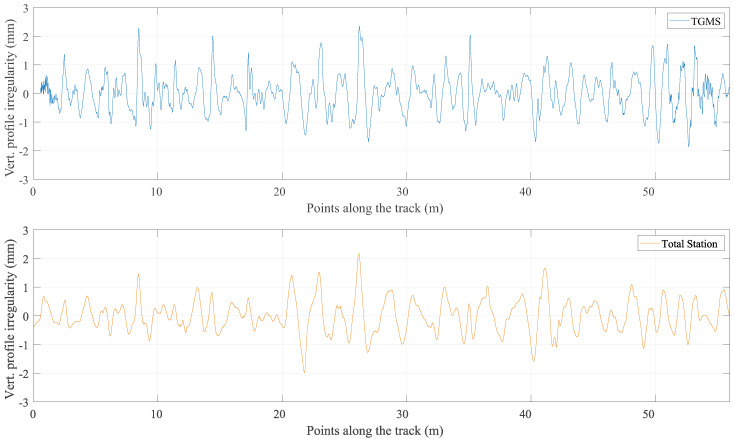
Measurements of vertical profile.

The results of this program are now compared with the reference value of the irregularities that were obtained with the method described in the previous section. Figure 16, Figure 17, Figure 18 and Figure 19 show the comparison between the reference geometry measurement explained in the previous section and the measurement of the TGMS developed in this investigation. The experiment was done with the vehicle shown in Figure 14 at a forward velocity of 2.5 m/s, that, considering the scale, it is equivalent to 25 m/s = 90 km/h of a real train. This is approximately the maximum forward velocity of metropolitan trains. The figures show the irregularities in the first 56 m of the track. The complete length of the scale track could not be measured because the vehicle had difficulties to negotiate the 12 m-radius curve at that forward velocity. The irregularities shown in the figures are high-pass filtered with a cut-off wave length of 3 m (wavelengths above 3 m are filtered out). It is a common practice in the geometry measurement of metropolitan trains to measure just wave lengths below 30 m, what is equivalent to 3 m in our scaled track.

The cross-level shown in Figure 16 shows a good agreement between the reference geometry and the measure of the TGMS. The agreement in gauge variation shown in Figure 17 is even better. Both curves are almost identical. However, in both figures, some edge effect is observed at the beginning of the measurement.

The absolute irregularities are compared in Figure 18 and Figure 19. As it can be observed, the agreement is fine, but not as good as the agreement of the relative track irregularities. The reason is clearly the influence in the results of the approximations and noise associated with the calculation of the TGMS to TF relative trajectory explained in Section 9. Recall that this relative trajectory is not needed for the calculation of the relative irregularities. It can also be observed that the measurements of the designed TGMS shows higher spatial-frequencies than the measurement with the total station. However, these higher frequencies must not be considered as noise, because the measuring method used with the total station can not detect high-frequency (short wavelength) irregularities.

## 12. Summary and Conclusions

This paper explains the numeric algorithms used to calculate track geometry irregularities with a new TGMS. These algorithms are basically kinematic relationships that describe the motion of a body moving along a track. After the description of the system and the sensors needed, the multibody-based kinematics is described in detail as well as the approximations used due to the small angles assumption. In this kinematic description, the track geometry (design geometry plus irregularities) is assumed totally arbitrary. As a result that the TGMS uses a computer vision system, the kinematic equations needed to locate the recorded rail cross-sections in the TGMS frame have been deduced, as well as a method to find the relative coordinates of the rail cross-sections with respect to the TGMS frame.

Section 6 shows the equations that can be used to find the value of the four independent track irregularities: gauge and cross-level in Equation (24), and alignment and vertical profile in Equation (27), as a function of the output of the computer vision system (explained in Section 5), the relative TGMS to TF relative orientation and the relative TGMS to TF relative trajectory. However, there is a long way to go to apply these equations.

Section 7 explains the development of a linear dynamic model that can be used to find the TGMS to TF relative orientation using the installed inertial sensors, 3D accelerometer and 3D gyroscope. This model is the basis of a Kalman filter that provides as the output the required TGMS angles. The application of this algorithm requires a precise knowledge of the position of the TGMS along the track. This position together with the vehicle forward velocity are needed to calculate the accelerations due to the motion of the TGMS along the design geometry of the track. In turn, these accelerations are needed to calculate the relative orientation based on the accelerometer signals. Section 8 explains the odometry algorithm that is used in this investigation for the precise calculation of the value of the arc-length *s* of the TGMS along the track. This algorithm is based on the measurement of the track horizontal curvature and the knowledge of the track design geometry.

The most difficult step for the calculation of the track irregularities is to obtain the TGMS to TF relative trajectory. Fortunately, this trajectory is needed to find the absolute irregularities but not needed to find the relative irregularities. The calculation of the relative trajectory is explained in Section 9. After a set of approximations, Equation (46) shows a second-order linear system of ordinary differential equations that can be solved to find the TGMS to TF relative trajectory. However, these equations are intrinsically unstable, requiring special solution methods that need to be improved, as it can be deduced from the experimental results.

The calculation of the track irregularities has been experimentally validated in a scale track that is described in Section 10 as well as the alternative method used to measure the track irregularities. This alternative method is accurate and based on the sensors used in the commercial rail track trolleys, LVDT and inclinometer, and a total station. The comparison of the measurements of the TGMS presented in this paper and this reference measurement is shown in Section 11. The relative irregularities are measured with high accuracy. The measurement of the absolute irregularities can be considered as acceptable, but the accuracy is much less. It can be concluded that the method of calculation of the TGMS to TF relative trajectory, that is the additional input needed for the calculation of the absolute irregularities, needs to be improved.

## Data Availability

The data presented in this study are available on request from the corresponding author.

## Abbreviations

The following abbreviations are used in this manuscript:

TGMS	Track geometry measurement system.
RTT	Rail track trolleys.
TRV	Track recording vehicles.
VMS	Versine measurement systems.
IMS	Inertial measurement systems.
<X,Y,Z>	Global frame (GF).
$<X^{t},Y^{t},Z^{t}>$	Track frame (TF).
$<X^{i},Y^{i},Z^{i}>$	Body frame *i* (BF).
$<X^{tgms},Y^{tgms},Z^{tgms}>$	TGMS frame.
$<X^{lrp},Y^{lrp},Z^{lrp}>$	Left rail-profile frame (LRP).
$<X^{rrp},Y^{rrp},Z^{rrp}>$	Right rail-profile frame (RRP).
$\vec{R}$	Position vector with respect to the GF.
${\vec{R}}^{t}$	Position vector of the TF with respect to the GF.
$\vec{r}$	Position vector with respect to the TF.
$\vec{u}$	Position vector with respect to the BF, LRP or RRP.
${\vec{u}}_{P}^{i}$	Position vector of point *P* that belongs to body *i*.
v	Components of a generic vector $\vec{v}$ in the GF.
$\bar{v}$	Components of a generic vector $\vec{v}$ in the TF.
$\hat{v}$	Components of a generic vector $\vec{v}$ in the BF, LRP or RRP.
$A^{t}$	Rotation matrix of the TF with respect to the GF.
$A^{i}$	Rotation matrix of the BF of body *i* with respect to the GF.
$A^{t,i}$	Rotation matrix from the BF of body *i* to the TF.
$\rho_{h}$, $\rho_{v}$, $\rho_{tw}$	Horizontal, vertical and twist curvatures of the track centerline.
${\rho_{h}}^{\prime }$	Spatial-derivative of horizontal curvature.
$\alpha_{v}$	Vertical slope of the track centerline.
$\psi^{t}$, $\theta^{\;t}$, $\varphi^{\;t}$	Euler angles that describe the orientation of the TF with respect to
	the GF.
$\psi^{i}$, $\theta^{\;i}$, $\varphi^{\;i}$	Euler angles that describe the orientation of the body *i* with respect to
	the TF.
$\psi_{abs}^{\;i}$, $\theta_{abs}^{\;i}$, $\varphi_{abs}^{\;i}$	Euler angles that describe the orientation of the body *i* with respect to
	the GF.
*s*	Arc-length coordinate.
*V*, $\dot{V}$	Forward velocity and acceleration.
${\vec{\omega }}^{t}$, ${\vec{\alpha }}^{t}$	Absolute angular velocity and acceleration vectors of the TF.
${\vec{\omega }}^{i}$, ${\vec{\alpha }}^{i}$	Absolute angular velocity and acceleration vectors of body *i*.
${\vec{\omega }}^{t,i}$, ${\vec{\alpha }}^{t,i}$	Angular velocity and acceleration vectors of body *i* with respect to
	the TF.
${\vec{r}}^{lir}$ = [ 0, $y^{lir}$, $z^{lir}$]	Irregularity vector of the left rail.
${\vec{r}}^{rir}$ = [ 0, $y^{rir}$, $z^{rir}$]	Irregularity vector of the right rail.
$\xi_{al},\xi_{vp},\xi_{gv},\xi_{cl}$	Alignment, vertical profile, gauge variation and cross level.
